# To explore the effect of kaempferol on non-small cell lung cancer based on network pharmacology and molecular docking

**DOI:** 10.3389/fphar.2023.1148171

**Published:** 2023-07-18

**Authors:** Junli Zhang, Xiangqi Liu, Guoying Zhang, Junling Wu, Zhongfang Liu, Chuanguo Liu, Hui Wang, Shuxin Miao, Lei Deng, Kuan Cao, Miwei Shang, Qingjun Zhu, Peng Sun

**Affiliations:** ^1^ Innovative Institute of Chinese Medicine and Pharmacy, Shandong University of Traditional Chinese Medicine, Jinan, China; ^2^ Experimental Center, Shandong University of Traditional Chinese Medicine, Jinan, China; ^3^ Department of Scientific Research, Shandong University of Traditional Chinese Medicine, Jinan, China; ^4^ Shandong Tai’an Central Hospital, Tai’an, China; ^5^ Key Laboratory of Traditional Chinese Medicine Classical Theory, Ministry of Education, Shandong University of Traditional Chinese Medicine, Jinan, China; ^6^ Shandong Provincial Key Laboratory of Traditional Chinese Medicine for Basic Research, Shandong University of Traditional Chinese Medicine, Jinan, China; ^7^ Daiyue District Maternal and Child Health Hospital, Tai’an, Shandong, China; ^8^ School of Pharmacy, Shandong University of Traditional Chinese Medicine, Jinan, China

**Keywords:** kaempferol (compound CID 5280863), network pharmacology, NSCLC, molecular docking, *in vitro* experiments

## Abstract

Non-small cell lung cancer (NSCLC) is a common pathological type of lung cancer, which has a serious impact on human life, health, psychology and life. At present, chemotherapy, targeted therapy and other methods commonly used in clinic are prone to drug resistance and toxic side effects. Natural extracts of traditional Chinese medicine (TCM) have attracted wide attention in cancer treatment because of their small toxic and side effects. Kaempferol is a flavonoid from natural plants, which has been proved to have anticancer properties in many cancers such as lung cancer, but the exact molecular mechanism is still unclear. Therefore, on the basis of *in vitro* experiments, we used network pharmacology and molecular docking methods to study the potential mechanism of kaempferol in the treatment of non-small cell lung cancer. The target of kaempferol was obtained from the public database (PharmMapper, Swiss target prediction), and the target of non-small cell lung cancer was obtained from the disease database (Genecards and TTD). At the same time, we collected gene chips GSE32863 and GSE75037 in conjunction with GEO database to obtain differential genes. By drawing Venn diagram, we get the intersection target of kaempferol and NSCLC. Through enrichment analysis, PI3K/AKT is identified as the possible key signal pathway. PIK3R1, AKT1, EGFR and IGF1R were selected as key targets by topological analysis and molecular docking, and the four key genes were further verified by analyzing the gene and protein expression of key targets. These findings provide a direction for further research of kaempferol in the treatment of NSCLC.

## 1 Introduction

Non-small cell lung cancer (NSCLC) is the leading cause of cancer death worldwide (Kumar, Yadavilli, & Kannan, 2021). Non-small cell lung cancer (NSCLC) accounts for 85% of lung cancer. NSCLC includes many cancer types, such as adenocarcinoma (LUAD), squamous cell carcinoma (LUSC) and large cell carcinoma. LUADs and LUSCs are the largest subsets of NSCLC (Relli, Trerotola, Guerra, & Alberti, 2019). Generally, NSCLC is treated by surgery in the early stage, but most NSCLC is found in the late stage. Therefore, at present, the treatment methods of NSCLC mainly include radiotherapy, chemotherapy, targeted therapy and immunotherapy, etc. However, radiotherapy, chemotherapy and targeted therapy will produce toxic side effects and drug resistance, and immunotherapy will also face the challenge of low response rate (C. Li, Qiu, & Zhang, 2022). Therefore, it is very important to find a natural extract with few side effects and clarify its molecular mechanism. These polyphenol compounds, such as kaempferol, curcumin, quercetin and resveratrol, which are natural plant extracts, have powerful anti-tumor effects. They can reverse epigenetic changes of oncogene activation and tumor suppressor gene inactivation, and are powerful anticancer agents (Rajendran et al., 2022). Kaempferol is a flavonoid, which exists in many vegetables, fruits, ginkgo biloba and so on (Rajendran et al., 2014). There are reports in the literature, Kaempferol can inhibit cell proliferation, motivation and invasion, and stimulate apoptosis and autophagy, consensus with modifications in coding and non-coding genes’ expression (Budisan et al., 2019). Studies have proved that kaempferol may induce NSCLC cell apoptosis through antioxidant pathway (Leung et al., 2007). Although kaempferol has a good application prospect in tumor therapy, there is little research on the molecular mechanism of kaempferol against non-small cell lung cancer, and there is a lack of systematic and complete understanding. In order to better understand the molecular mechanism of drug anti-tumor, more and more scholars use network pharmacology for research. Pharmacology of the network is the cross synthesis of pharmacology, system biology, bioinformatics, network science and other disciplines, which can systematically explain the mechanism of action of drugs (X. Wang, Hu, Zhou, & Li, 2022). With the development of bioinformatics, some high-throughput platforms are usually used to analyze gene expression during tumorigenesis, which is of great significance in revealing the molecular mechanism of diseases (L. Wang et al., 2020). On the basis of experimental verification, with the help of network pharmacology, molecular docking and bioinformatics, this study predicted the molecular mechanism of kaempferol’s intervention in non-small cell lung cancer.

## 2 Materials and methods

### 2.1 Cell culture and cell viability detection

At first, CCK-8 kit was used to evaluate the effect of kaempferol on the viability of BEAS-2B cells. BEAS-2B cells were seeded in 96-well plates at 5 * 10 ^ 3 cells per well and cultured in 37°C and 5% CO2 incubator for 24 h. Complete culture medium (blank control), 0.1% DMSO (negative control),10 μg/mL cisplatin (DDP positive control) and KA (10 μM, 50μM and100 μM) were given respectively, and the culture continued for 48 h. Then, the culture medium was removed, and 100 μL of fresh culture medium containing 10%CCK-8 was added to each well and cultured for 2 h. OD value at 450 nm was measured by microplate reader. Three independent experiments were conducted. Next, CCK-8 kit was used to evaluate the effect of kaempferol on the viability of A549 cells. Three independent experiments were conducted.

### 2.2 Scratch experiment

Lung cancer A549 cells were inoculated into 24-well plates with 2 * 10 4 cells per well, and incubated overnight in 37°C and 5% CO2 incubator. Scribe vertically with 200 μL pipette tip, and then wash with PBS for 3 times. The cells were treated with complete culture medium (blank control), 0.1% DMSO (negative control), 10 μg/mL cisplatin (DDP positive control) and 50 μM kaempferol, and each group had three accessory holes, and continued to be cultured for 48 h. The migration distance of cells was measured and recorded by microscope. Three independent experiments were conducted.

### 2.3 Transwell invasion experiment

Serum-free medium was used to dilute the matrix glue at the ratio of 1:8, and 100 μL was added to the upper chamber, which was placed in an incubator at 37°C. When the matrix glue solidified, 200 μL of cell suspensions treated with different drugs (complete medium, 0.1% DMSO, 10 μg/mL cisplatin, 50 μM kaempferol) were added to the upper chamber, with 2 * 10^4 cells per well. Add 600 μL complete medium (containing 10% FBS) into the lower chamber. Lung cancer A549 cells were cultured in an incubator for 48 h. Then suck out the culture medium in the upper chamber, clean the remaining cells in the upper chamber with cotton swabs, and wash them twice with PBS. Soak in 4% paraformaldehyde for 20 min, air dry, dye with 1% crystal violet solution for 15 min, and wash with PBS for 3 times. The adherent cells were observed and counted by microscope. Three independent experiments were conducted.

### 2.4 Collect kaempferol targets

Pubchem (https://pubchem.ncbi.nlm.nih.gov/) the kaempferol Canonical SMILES obtained are imported into SwissTargetPrediction (https://www.swisstargetprediction.ch/), to obtain the target with *p* > 0; The target of kaempferol was obtained by Pharm Mapper (https://www.lilab-ecust.cn/pharmmapper/). The acquired target gene set was imported into UniprotKB (https://www.uniprot.gov/) database for standardization.

### 2.5 Acquisition of NSCLC target gene

Search target genes of non-small cell lung cancer from GeneCards database (https:// www.genecards.org/) and TTD database (https://db.idrblab.net/ttd/). The target gene was integrated and de-duplicated. Selected and downloaded the data of GSE32863 and GSE75037 chips from the GEO database (https://www.ncbi.nlm.nih.gov/). Gene chip data came from the same annotation platform, and the sample species were *Homo sapiens*, including tumor group and control group. The differential genes were screened by Limma package in R software, with ∣Log2FC∣≥1 and correction *p* < 0.05; Using pheatmap and ggplot2 packages to draw the heat map and volcano map of different genes.

### 2.6 Screening of core targets of kaempferol in the treatment of non-small cell lung cancer

In order to determine the interaction target of kaempferol in the treatment of NSCLC, we imported kaempferol prediction target gene and non-small cell lung cancer target gene into Venny2.1.0 (https://bioinfogp.cnb.csic.es/tools/venny/) online website to obtain the intersection gene and draw the Wayne diagram. Introducing intersection targets into STRING online platform to construct protein interaction network. Set the species source as *H. sapiens*, and the confidence level as medium confidence level. The results were imported into Cytoscape3.9.0 software to build PPI network, and the core targets were screened out by CytoNCA plug-in. Select degree centrality, closeness centrality and betweenness centrality in CytoNCA plugin to screen core targets.

### 2.7 Enrichment analysis of GO and KEGG

The intersection targets were imported into DAVID database (https://david.ncifcrf.gov/) for GO and KEGG enrichment analysis respectively, and some results were visualized by drawing bubble chart and chord chart with bioinformatics online tool (https://vip.sangerbox.com/login.html).

### 2.8 Molecular docking between kaempferol and core targets

In order to understand the mechanism of action between kaempferol and core targets, molecular docking was carried out. The 2D structure of kaempferol downloaded from PubChem database was imported into Chem Bio3D Ultra 14.0 for correction, and stored in mol2 format. Download the core target protein from PDB database. SYBYL was used to dehydrate and hydrogenate the core target protein, and then it was molecular docked with kaempferol, and some results were visualized.

### 2.9 Expression level of core target genes

We used GEPIA (https://gepia.cancer-pku.cn/) database to analyze the differential expression of core targets between tumor tissues and normal tissues. The data comes from TCGA and GTEx databases.

### 2.10 Protein expression level of core target

HPA (https://www.proteinatlas.org/) Database uses transcriptomics and protein omics techniques to study protein expression in different human tissues and organs at RNA and protein levels. According to the staining intensity and the percentage of stained cells in tissues, we compared the protein expression levels of core genes in NSCLC tissues and normal lung tissues, and selected representative immunohistochemical staining pictures from HPA database.

### 2.11 Survival analysis

Kaplan-Meier Plotter Database (https://kmplot.com/analysis/) is an online analysis database related to the prognosis of malignant tumors, which can be used to explore the relationship between target gene expression and the prognosis of related tumors. Therefore, we use this database to draw the survival curve of key genes, and analyze the influence of the expression of key genes in NSCLC on the overall survival of patients.

## 3 Results

### 3.1 The effect of kaempferol on A549 cell viability

**FIGURE 1:**
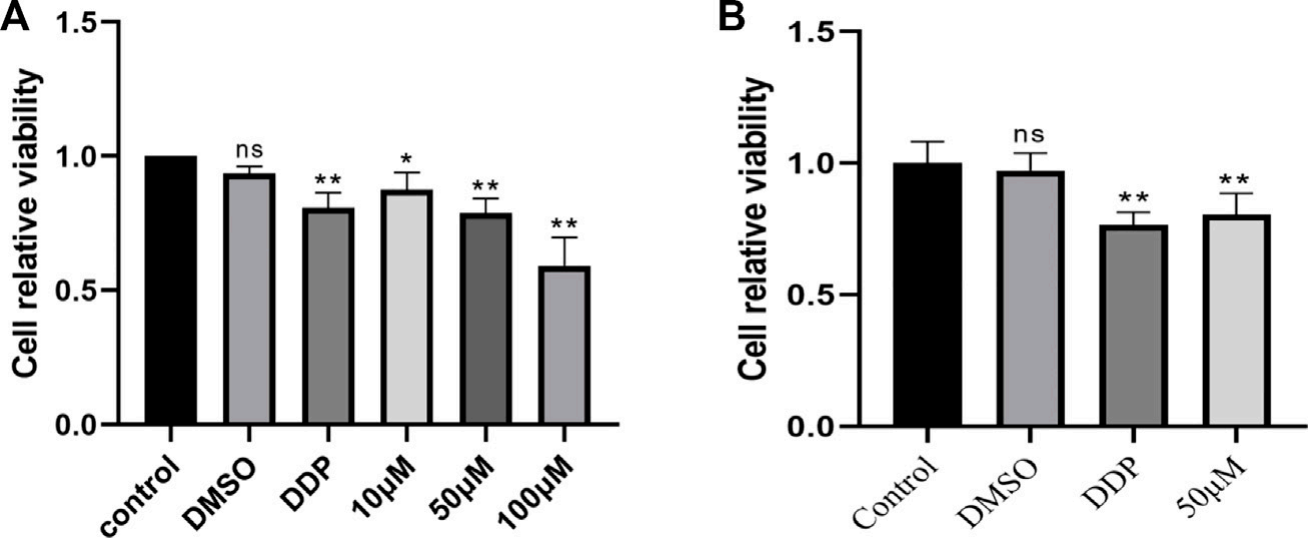
**(A)** Cell viability of BEAS-2B cells treated with complete medium, 0.1% DMSO, 10 μg/mL cisplatin and KA (10 μM, 50 μM and 100 μM) for 48 h was measured by CCK-8. **(B)** Cell viability of A549 cells treated with complete medium, 0.1% DMSO, 10 μg/mL cisplatin and KA50 μM for 48 h was measured by CCK-8. (* Compared with the control group, *p* < 0.05, * * compared with the control group, *p* < 0.01).

CCK-8 assay was used to detect the cell viability of BEAS-2B cells treated with complete medium, 0.1% DMSO, 10 μg/mL cisplatin and KA (10 μM, 50 μM, and 100 μM) for 48 h. The experimental results showed that (Figure 1), when the concentration was 10 μM and 50 μM, KA had less toxicity to cells. Therefore, we chose 50 μM to act on A549 cells for 48 h. The experimental results showed that (Figure 1), compared with the blank control group and the negative control group, kaempferol had a significant inhibitory effect on the proliferation of lung cancer A549 cells.

### 3.2 Effect of kaempferol on migration and invasion of A549 cells

**FIGURE 2:**
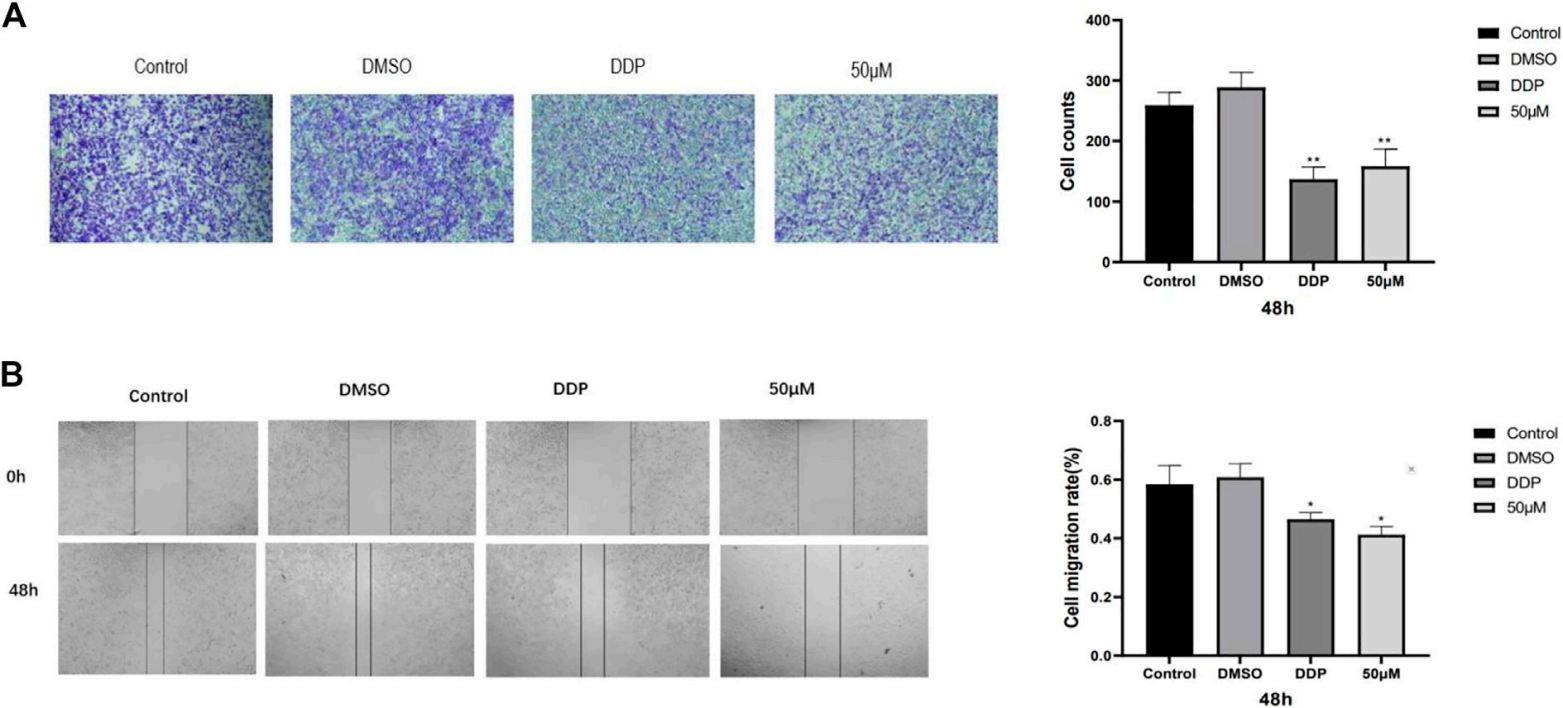
**(A)** Image of cell migration process in scratch experiment and 48-h cell migration rate. **(B)** Cell invasion experiment. Statistics of cell invasion images and cell invasion counts after 48 h of treatment in different drug groups. (* Compared with the control group, *p* < 0.05, * * compared with the control group, *p* < 0.01).

To study the effect of kaempferol on the migration and invasion of A549 cells, scratch test and invasion test were carried out. The results showed that compared with the control group, kaempferol could significantly inhibit the migration of A549 cells, and the effect was slightly better than that of the positive control group. In the invasion experiment, compared with the control group, kaempferol can significantly inhibit the invasion of lung cancer A549 cells. (Figure 2).

### 3.3 Kaempferol and NSCLC targets

**FIGURE 3:**
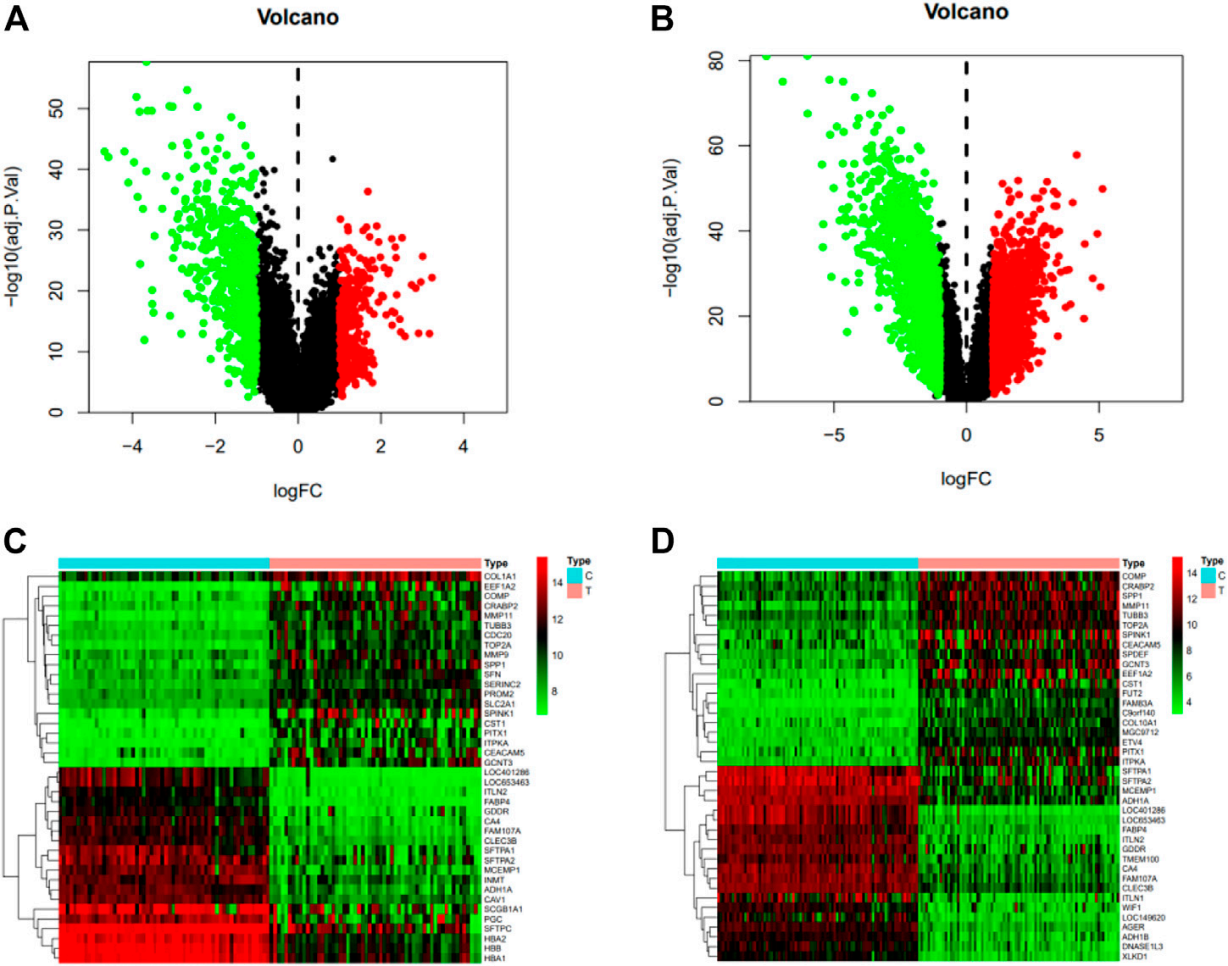
**(A)** Volcano map of GSE32863 differential genes, with red representing upregulated genes (logFC > 0), green representing downregulated genes (logFC < 0) and black representing no significant difference. **(B)** Volcano map of GSE75037 differential genes, with red representing upregulated genes (logFC > 0), green representing downregulated genes (logFC < 0) and black representing no significant difference. **(C)** The heat map of the first 40 differentially expressed genes in GSE32863, with red indicating upregulation, green indicating downregulation, and black indicating no significant difference. **(D)** Heat map of the first 40 differentially expressed genes of GSE75037.

According to the screening conditions, we got 113 predicted targets of kaempferol. After de-duplication of two disease databases, we got 5818 targets of non-small cell lung cancer. 956 and 2856 differential genes were obtained from gene chips GSE32863 and GSE75037, respectively. There are 339 upregulated genes and 617 downregulated genes in GSE32863, and 1313 upregulated genes and 1543 downregulated genes in GSE75037. According to the two data sets, a volcano map is constructed (Figure 3). We selected the first 40 differential genes to make a gene heat map (Figure 3).

### 3.4 Kaempferol is the core target of NSCLC

**FIGURE 4:**
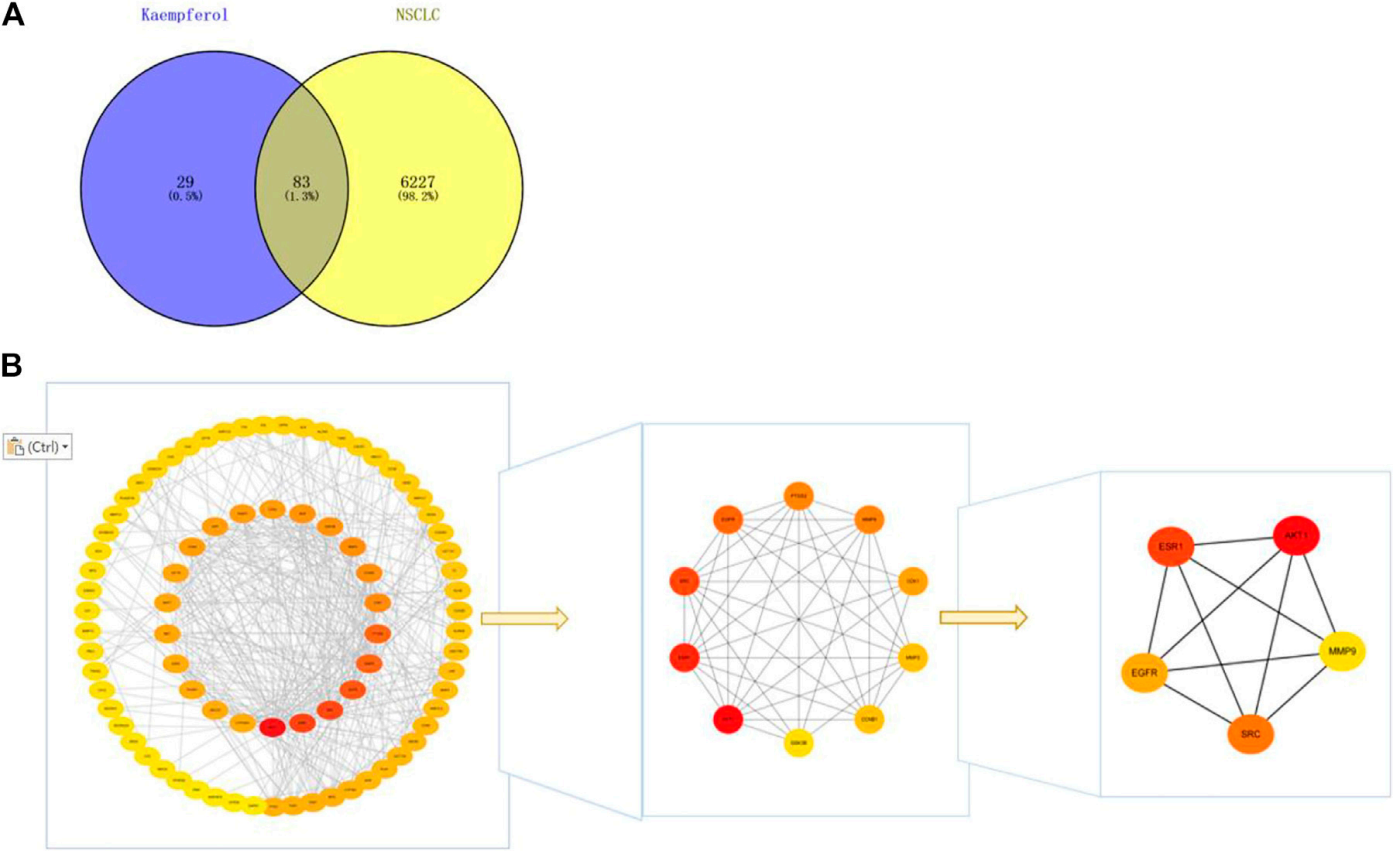
**(A)** Wayne diagram of kaempferol target and NSCLC target, with a total of 83 intersecting targets. **(B)** Get the top five core targets from PPI network by using cytohubba plug-in.

By drawing the Wayne diagram (Figure 4), we got 83 intersecting targets of kaempferol and NSCLC, and identified them as potential candidate targets of kaempferol in the treatment of NSCLC. Then, we import the candidate targets into the STRING database to obtain the protein-protein interaction network. We imported the PPI network into Cytoscape for visualization, and determined AKT1, ESR1, SRC, EGFR, and MMP9 as targets for further analysis according to the degree, closeness and betweenness value in the CytoNCA plug-in (Figure 4).

### 3.5 Enrichment analysis of Go and KEGG

**FIGURE 5:**
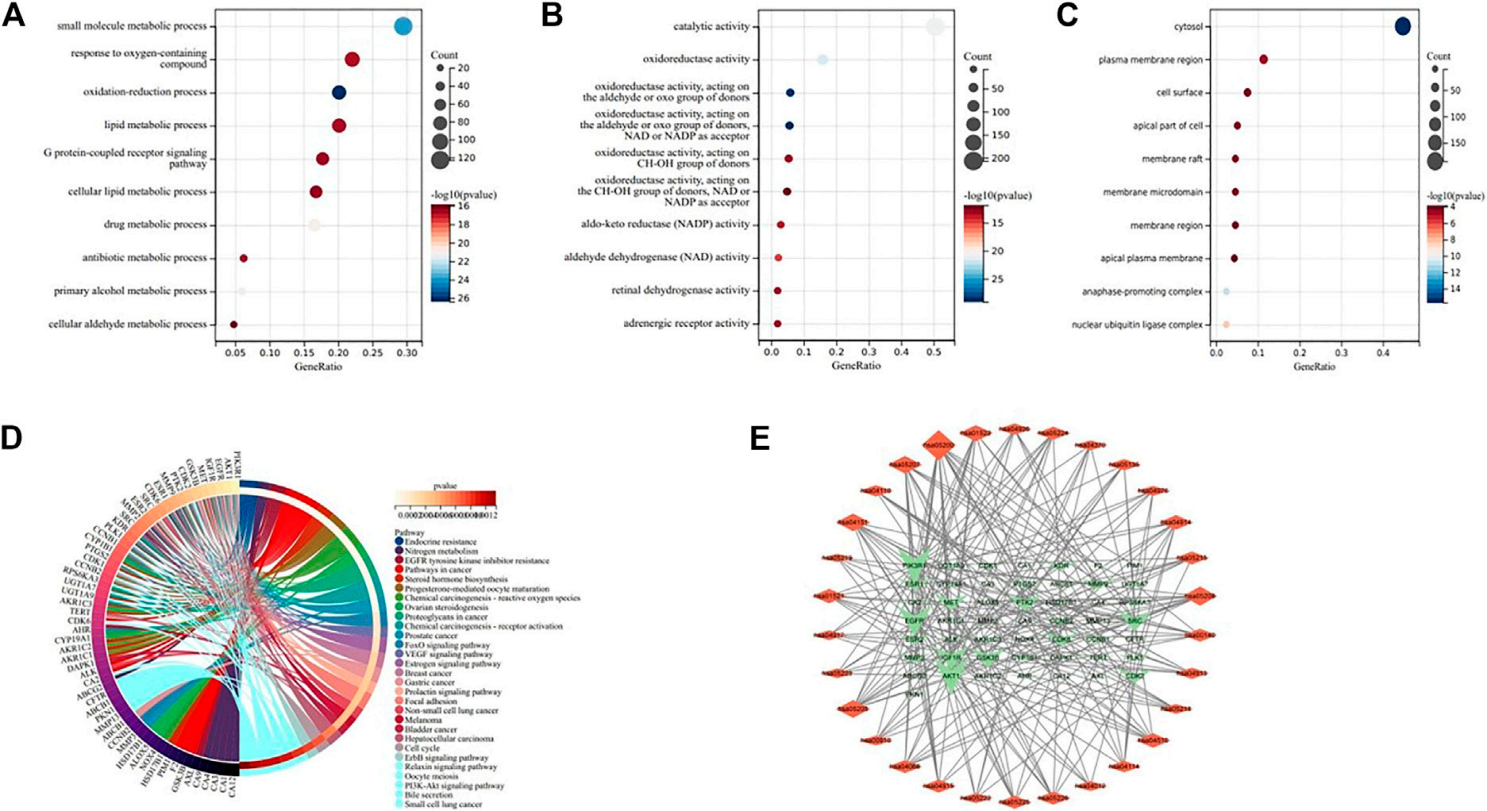
**(A–C)** Bubble diagram of GO enrichment analysis, indicating BP, MF, and CC respectively. **(D)** KEGG enrichment analysis chord diagram. **(E)** “Pathway-Target” network diagram. The network shows the relationship between the first 30 pathways and 50 genes, and the size of the graph shows the number of connected pathways and genes.

The results of GO analysis (Figure 5) clarify the biological process, molecular function and cell components. The enrichment analysis showed that the biological processes mainly included peptidyl-serine phosphorylation, protein autophosphorylation, protein phosphorylation, negative regulation of apoptotic process, positive regulation of protein kinase B signaling and other processes related to cell apoptosis. The molecular functions were related to cell activity, such as protein kinase activity, ATP binding, protein serine/threonine kinase activity, carbonate dehydratase activity, enzyme binding. The cell components were related to the cyclin-dependent protein kinase enzyme complex, cytosol, macromolecular complex, nucleus and plasma membrane. The results of KEGG enrichment analysis showed that there were 54 pathways with *p* < 0.01, and the first 30 pathways were selected to draw chords for visualization (Figure 5). Among them, the main gene-rich pathways include Endocrine resistance, Nitrogen metabolism, EGFR tyrosine kinase inhibitor resistance, Pathways in cancer, Steroid hormone biosynthesis and so on, and we found that these signal pathways are closely connected with PI3K/Akt. We also found that the core targets were closely related to the PI3K/Akt signaling pathway. To verify this finding, we made a “pathway-target” network diagram. In the “pathway-target” (Figure 5) network diagram, the genes associated with the most pathways are PIK3R1, AKT1, EGFR, IGF1R and SRC, and the signaling pathway associated with the most targets is PI3K/Akt. Based on the above results, we speculate that the key targets of kaempferol in NSCLC may be PIK3R1, AKT1, EGFR, IGF1R and SRC, and the possible signaling pathway is PI3K/Akt.

### 3.6 Molecular docking

**FIGURE 6:**
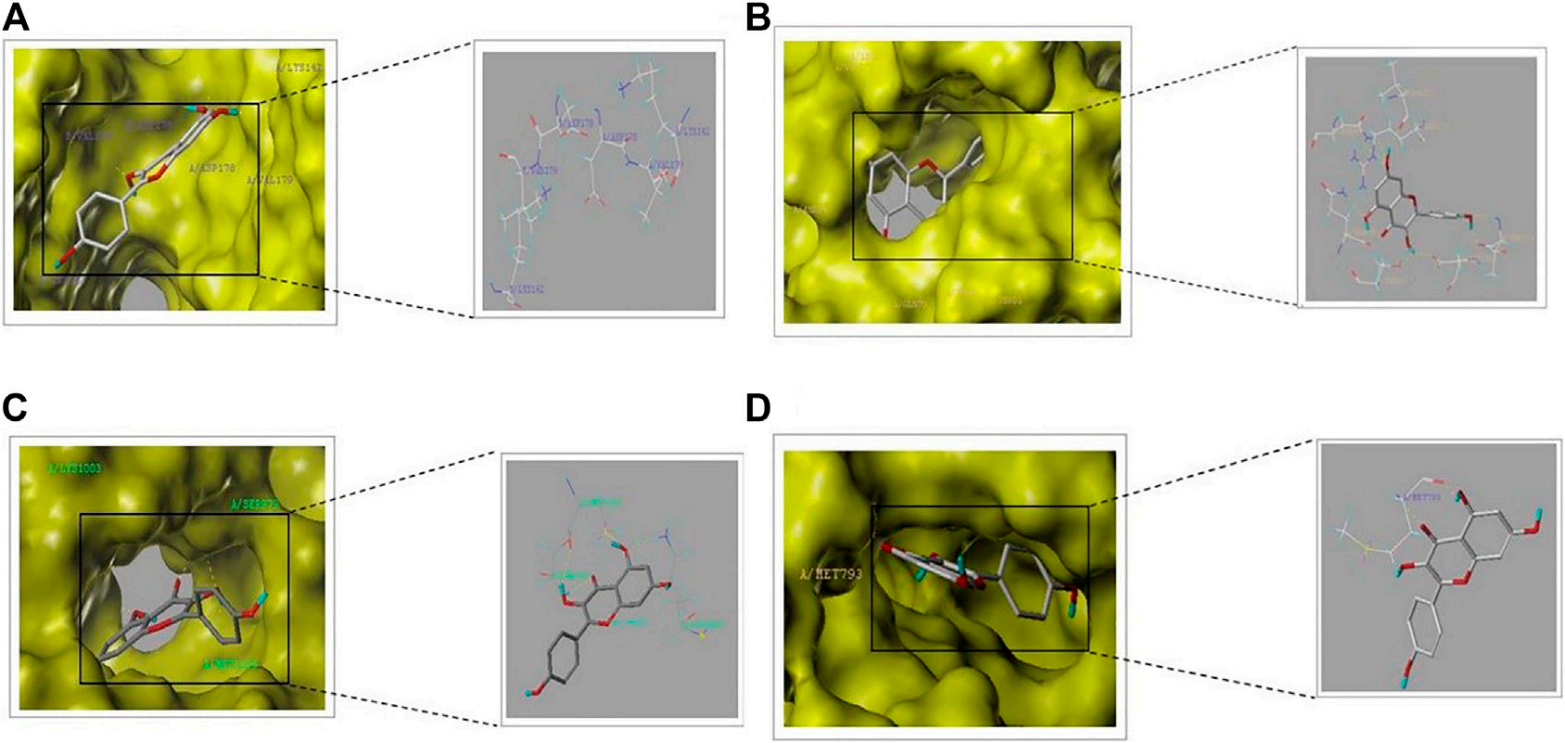
Schematic diagram of molecular docking part: **(A)** PIK3R1-kaempferol. **(B)** AKT1-kaempferol **(C)** IGF1R-kaempferol **(D)** EGFR-kaempferol.

In order to verify the reliability of kaempferol acting on core targets and interfering with key signaling pathways, we performed molecular docking on targets screened by CytoNCA and targets screened by “target-pathway”network diagram. In general, if the docking score > 5, it indicates that the molecules and proteins have good docking activity ; if the docking result > 7, it is considered to have strong docking activity (Jain, 2003). The molecular docking results show (Table 1) that among the seven targets, only PIK3R1, AKT1, EGFR and IGF1R have good docking activity with kaempferol (Figure 6), so we identified them as key core targets and further analyzed them.

**TABLE 1:** Molecular docking scores of kaempferol and core target protein.

Receptor	PDB id	Total score
PIK3R1	1PBW	5.84
AKT1	5KCV	5.67
EGFR	1XKK	5.56
IGF1R	2OJ9	5.69
ESR1	1HCP	3.15
SRC	1A08	3.95
MMP9	4XCT	4.02

### 3.7 Differential expression level of core target genes

**FIGURE 7:**
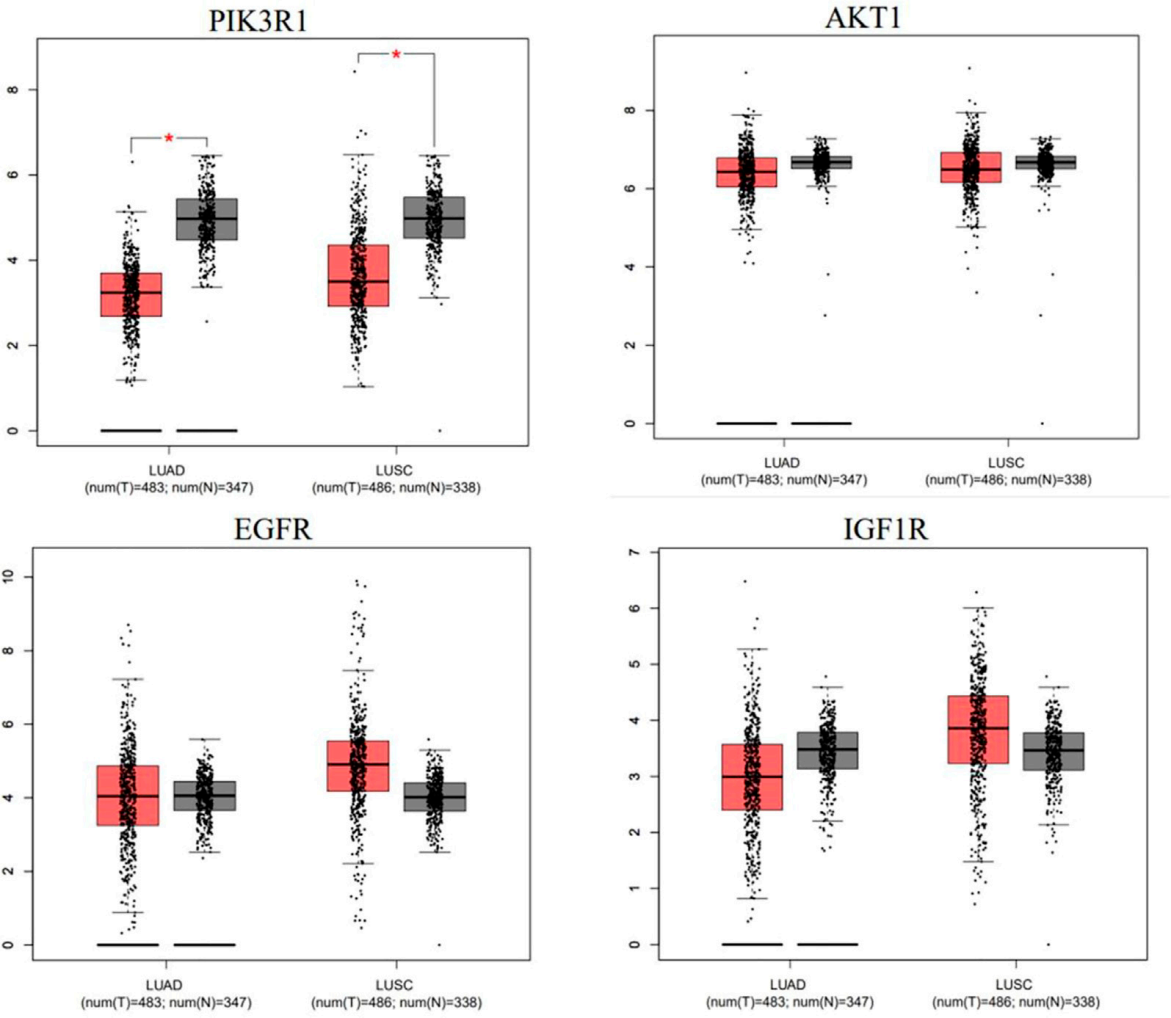
mRNA expression levels of hub genes in cancer genome map (TCGA) and genotypic tissue expression (GTEx) databases. Red represents lung cancer tissue, and black represents normal lung tissue.

We used GEPIA online web analytics to detect the differential expression of key core target genes between NSCLC tissues and normal lung tissues (Figure 7). The results showed that the mRNA expressions of AKT1, EGFR and IGF1R were upregulated in lung cancer tissues, while the expression of PI3KR1 was downregulated compared with normal lung tissues. These results suggest that the gene expression levels of these four core targets may be related to the progress of NSCLC.

### 3.8 Expression level of core target protein

**FIGURE 8:**
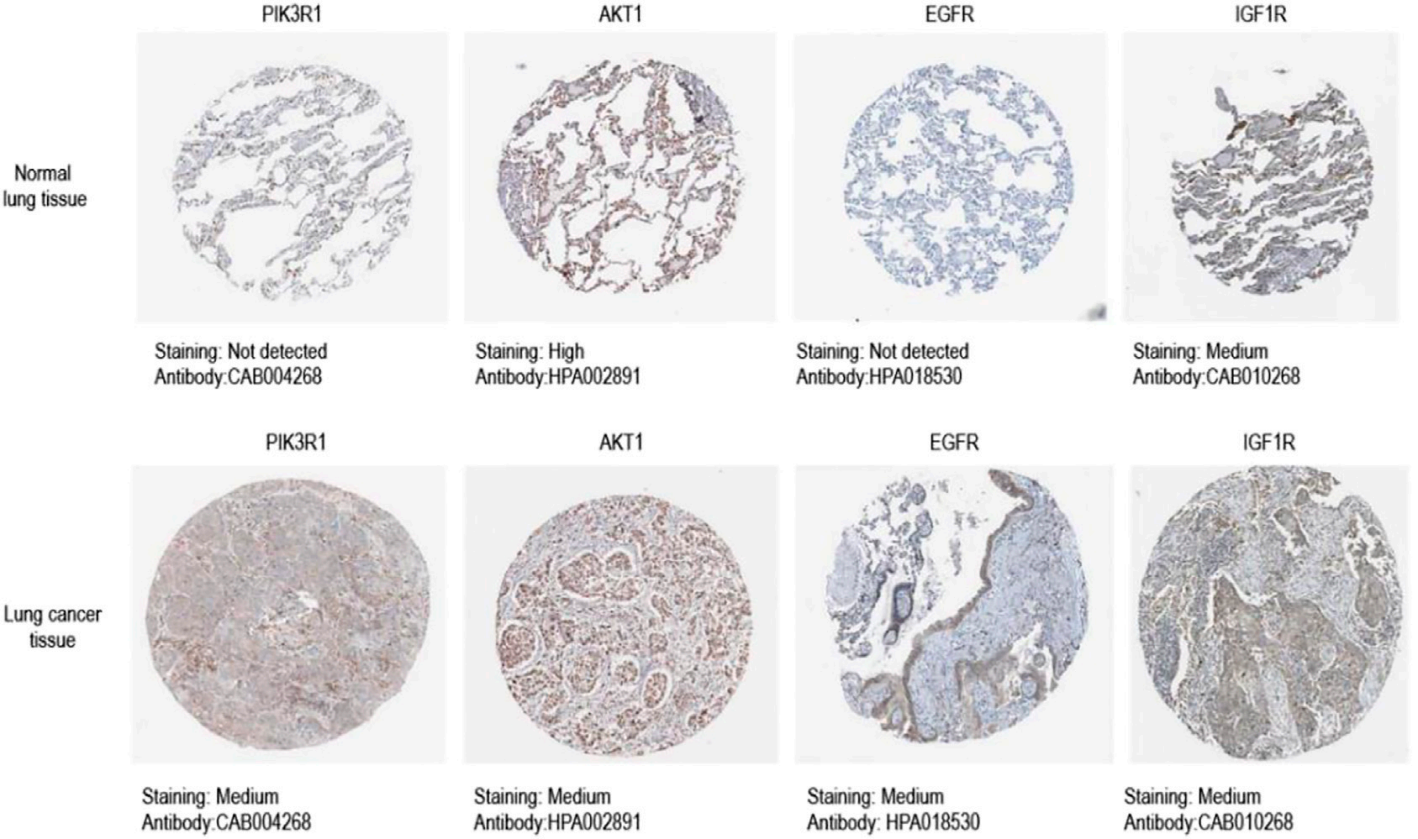
Immunohistochemical image of core target protein expression level in HPA database.Kaplan-Meier analysis of four key genes, PIK3R1, AKT, EGFR and IGF1R, in lung cancer patients.

We obtained immunohistochemical staining images of core targets in lung tumor tissues and normal lung tissues from HPA database to observe the protein expression level of core targets. As shown in the results (Figure 8), the expression level of all the core proteins in lung tumor tissues was moderate. It is suggested that the protein expression levels of these four core targets may be related to the progress of NSCLC.

### 3.9 Survival analysis

**FIGURE 9:**
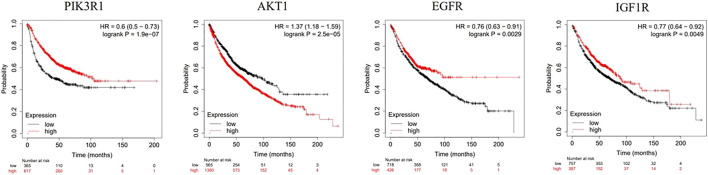
Kaplan-Meier analysis of four key genes, PIK3R1, AKT, EGFR and IGF1R, in lung cancer patients.

Survival analysis of four key genes, PIK3R1, AKT, EGFR and IGF1R (Figure 9), showed that the expression of these four key genes was significantly correlated with the prognosis of patients (*p* < 0.05).

## 4 Discussion

According to the global cancer statistics in 2020, at present, lung cancer is the second most common malignant tumor among new malignant tumor cases in the world, accounting for 11.4% of all cases. It is also the “number one killer” of cancer deaths, accounting for 18.0% of the total cancer deaths 18.0% (Sung et al., 2021). NSCLC, as the most common type of lung cancer, has a high metastasis rate and mortality, which poses a serious threat to the survival of the patients (Ahlawat, Phutela, Bal, Singh, & Sharma, 2022). Therefore, it is of great significance to study the molecular mechanism of the occurrence and development of NSCLC and explore effective therapeutic drugs and schemes for preventing the occurrence of NSCLC and improving the prognosis of NSCLC patients. As a natural plant extract, kaempferol is a flavonoid, which has been proved to exert anticancer properties in various cancers through different modes of action (Chen & Chen, 2013; Han, Yu, Yang, Shen, & Wang, 2017; Zhu & Xue, 2019) And compared with standard chemotherapy drugs, the toxicity to normal cells is much smaller (Zhang, Chen, Li, Chen, & Yao, 2008). At the same time, some literatures have pointed out that kaempferol can inhibit the growth of lung cancer A549 cells, induce cell apoptosis, and limit cell migration and invasion (Imran et al., 2019). In this study, the potential molecular mechanism of kaempferol against NSCLC was preliminarily discussed based on network pharmacology, molecular docking, and bioinformatics, on the basis of verifying the efficacy *in vitro*. First, we verified that kaempferol can effectively inhibit the proliferation, migration and invasion of lung cancer A549 cells through CCK-8 experiment, scratch experiment and invasion experiment. Then, we predicted and screened kaempferol targets with SwissTargetPrediction and PharmMapper database, obtained NSCLC targets with GeneCards, TTD and GEO database, and obtained cross targets with Wayne platform. Then, we build PPI network, visualize it through Cytoscape3.9.1, and use its plug-in SytoHbba to screen and identify the targets for further analysis AKT1, ESR1, SRC, EGFR, MMP9; Next, Go and KEGG enrichment analysis was performed on the cross targets to construct a “pathway-target” target network. The genes related to the most pathways were PIK3R1, AKT1, EGFR, IGF1R and SRC, and the signal pathway related to the most targets was PI3K/Akt. Seven targets AKT1, PIK3R1, ESR1, SRC, EGFR, MMP9 and IGF1R were verified by SYBYL software, and the targets AKT1, PIK3R1, EGFR and IGF1R with good docking effect were obtained. The gene differential expression analysis, protein expression analysis and survival analysis of these four core targets were carried out. The results showed that, The expression levels of genes and proteins of these four core targets may be related to the progress of NSCLC, and they are also involved in the whole survival process of NSCLC patients, and have reliable correlation, which may prove to be potential biomarkers for clinical detection and treatment of NSCLC. Therefore, we predict that kaempferol may regulate PI3K/AKT signaling pathway by targeting PIK3R1, AKT1, EGFR and IGF1R genes, and inhibit the proliferation, invasion and migration of NSCLC cells.

PI3K/AKT signaling pathway is an important carcinogenesis pathway. The activation of this pathway can promote the growth, survival, migration and invasion of tumor cells by affecting tumor cell cycle, inhibiting tumor cell apoptosis, autophagy, promoting tumor angiogenesis and chemotherapy resistance, etc. The activation of this pathway is often found in NSCLC (Chin et al., 2018; Liao et al., 2019). Kaempferol can inhibit tumor angiogenesis by regulating PI3K/AKT, MEK and ERK (Chin et al., 2018). Kaempferol can also affect the growth of NSCLC, colorectal cancer and cervical cancer cells by regulating PI3K/AKT signaling pathway (Imran et al., 2019; Kashafi, Moradzadeh, Mohamadkhani, & Erfanian, 2017; Q; Li et al., 2019). PIK3R1 is the regulatory subunit of phosphoinositide 3 kinase (PI3K), which participates in the biological processes of various human malignant tumors (Yuan & Cantley, 2008). It is a potential upregulated oncogene in NSCLC, and plays an important role in the inhibition of the growth of NSCLC cells by miR-486-5p (Tian et al., 2019) AKT is a serine/threonine protein kinase, which is an important downstream effector of PI3K signal. Overactivation of PI3K leads to the accumulation of AKT, and AKT is sensitive to the level of epidermal growth factor (EGFR) (Nomura et al., 2003; Chin et al., 2018). AKT1, as a subtype of AKT, may play an important role in the occurrence and development of non-small cell lung cancer (Zhou et al., 2019). Kaempferol Suppresses Transforming Growth Factor-β1–Induced Epithelial-to-Mesenchymal Transition and Migration of A549 Lung Cancer Cells by Inhibiting Akt1-Mediated Phosphorylation of Smad3 at Threonine-179 (Jo, Park, Choi, Jeon, & Kim, 2015). Epidermal growth factor receptor (EGFR) is a 170 kDa receptor tyrosine kinase (RTK). Phosphorylation of EGFR can activate phosphatidylinositol 3 kinase (PI3K), which activates downstream signal molecules in the pathway, thus regulating tumor cell proliferation, invasion, migration and apoptosis (Y. Liu et al., 2020). Inhibition of EGFR/PI3K/Akt signaling pathway can inhibit the proliferation, invasion and migration of NSCLC cells (Sato et al., 2018). Shihua Yao et al. pointed out that kaempferol has a direct effect on the activity of epidermal growth factor receptor (EGFR), while inhibiting EGFR, its downstream signaling pathway is also obviously inhibited. And confirmed kaempferol inhibitions cell proliferation and glycilysis in esophagus squamus cell carcinogena via targeting EGFR signaling pathway (Yao et al., 2016). Insulin-like growth factor 1 receptor (IGF1R) is a transmembrane receptor tyrosine kinase receptor and the upstream regulator of Akt. It is highly expressed in a variety of cancers such as lung adenocarcinoma, pancreatic cancer and breast cancer (Baserga, Peruzzi, & Reiss, 2003; Allen et al., 2007). Generally speaking, IGF1R will be activated by a specific ligand in the extracellular space (for example, IGF-I, IGF-II) (Durai, Yang, Gupta, Seifalian, & Winslet, 2005; Kim, Cho, Paik, & Kim, 2012) Then it will activate downstream PI3K/AKT signaling pathways, and play a vital role in cell proliferation and differentiation and anti-apoptosis (Bertrand et al., 2006; C. H; Liu et al., 2013). Hyun Sook Lee et al. proved that Kaempferol down regulations activation of PI3K/Akt and ERK-1/2 pathways by inhibiting IGF-IR and erbb3 signaling in HT-29 cells (Lee, Cho, Kwon, & Park, 2014).

In a word, EGFR, IGF1R, PIK3R1 and AKT are all expressed in different degrees in NSCLC. Kaempferol may regulate EGFR/PI3K/AKT and IGF1R/PI3K/AKT signaling pathways by targeting EGFR, IGF1R, PIK3R1 and Akt, and then play a role in the treatment of non-small cell lung cancer.

## 5 Conclusion

To sum up, this study verified that kaempferol can inhibit the proliferation, migration and invasion of lung cancer A549 cells based on *in vitro* experiments, and verified the pharmacological characteristics of kaempferol with multiple targets and multiple pathways based on network pharmacology, molecular docking and bioinformatics. It was predicted that Kaempferol may regulate EGFR/PI3K/AKT and IGF1R/PI3K/AKT signaling pathways by targeting EGFR, IGF1R, PIK3R1 and Akt1, which has certain guiding significance for the following experimental research and clinical research. Although there are some limitations in network pharmacology and molecular docking, it points out the direction for our subsequent experimental mechanism research.

## Data Availability

Publicly available datasets were analyzed in this study. This data can be found here: Pubchem (https://pubchem.ncbi.nlm.nih.gov/) Swiss Target Prediction (https://www.swisstargetprediction.ch/) Pharm Mapper (https://www.lilab-ecust.cn/pharmmapper/). UniprotKB (https://www.uniprot.gov/) Gene Cards database and TTD database, GEO database, GEPIA (https://gepia.cancer-pku.cn/), HPA (https://www.proteinatlas.org/), Kaplan-Meier Plotter Database (https://kmplot.com/analysis/).
