# Supplementary material for: To explore the effect of kaempferol on non-small cell lung cancer based on network pharmacology and molecular docking
Source: Front Pharmacol. 2023 Jul 18;14:1148171. doi: 10.3389/fphar.2023.1148171 (PMC10392700; doi:10.3389/fphar.2023.1148171)

1.CCK-8

(A)BEAS-2B cells

concentrati	48h		
control	1	1	1
DMSO	0.962393163	0.913940256	0.934483512
DDP	0.866239317	0.757467994	0.80541603
10μM	0.922649573	0.806543385	0.903035597
50μM	0.826495727	0.726173542	0.813933173
100μM	0.694444445	0.597439545	0.482419742

(B)A549 cells

concentrati	48h		
Control	0.91525577	1.077563129	1.007181101
DMSO	0.917046523	1.04614355	0.952698792
DDP	0.767111642	0.814810795	0.720226467
50μM	0.775088633	0.896479994	0.745134216

2.Invasion experiments

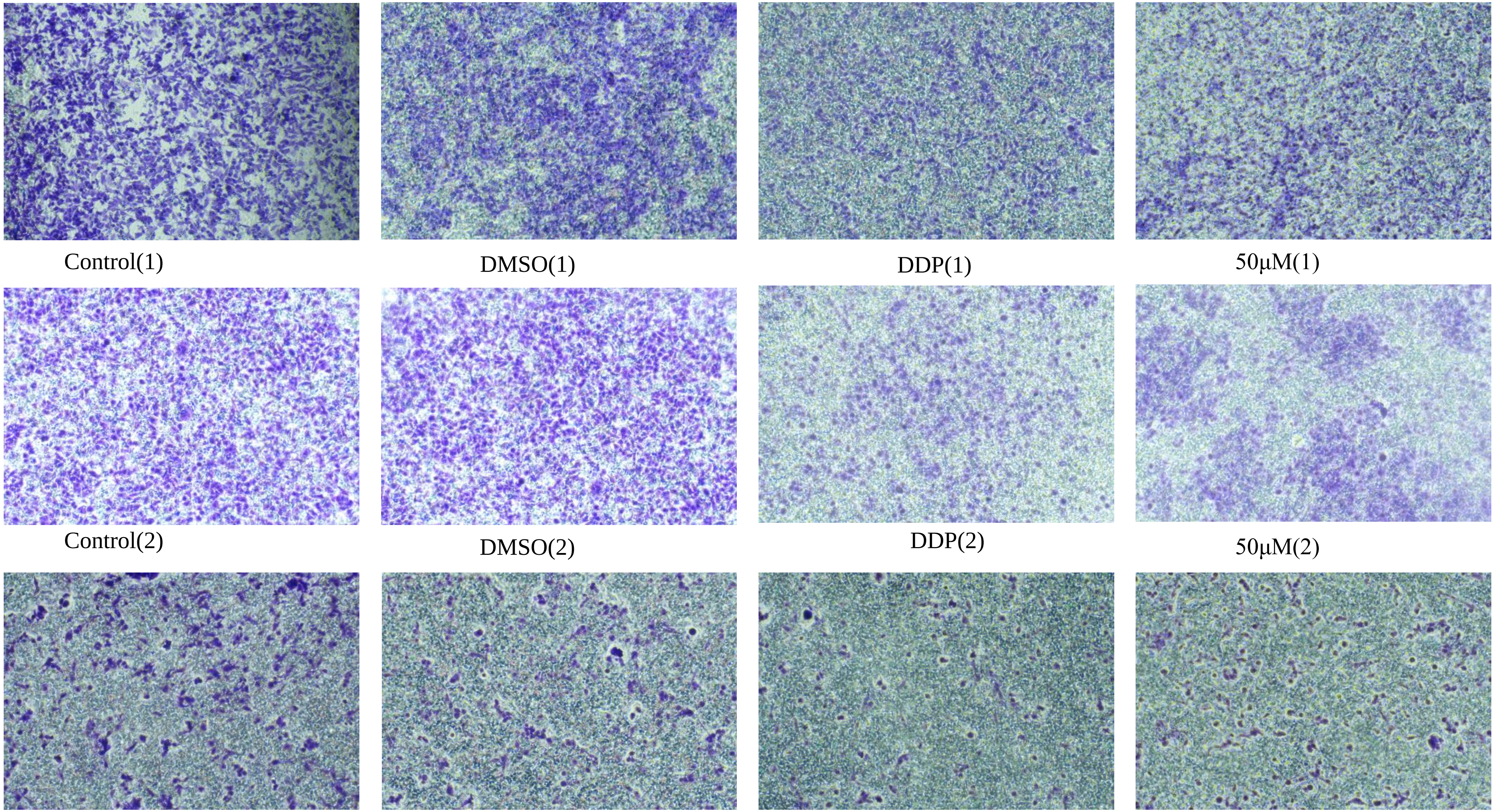

Control(1) DMSO(1) DDP(1) 50μM(1)

Control(2) DMSO(2) DDP(2) 50μM(2)

Control(3) DMSO(3) DDP(3) 50μM(3)

3.Wound Healing

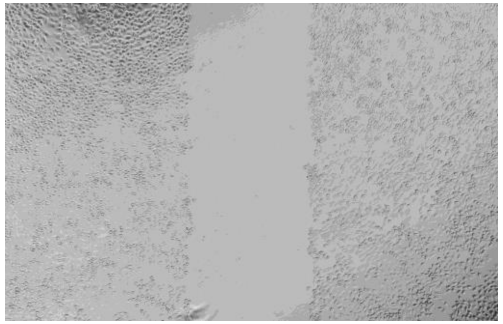

Control(1)

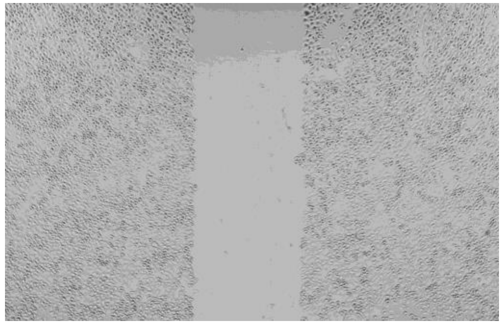

DMSO(1)

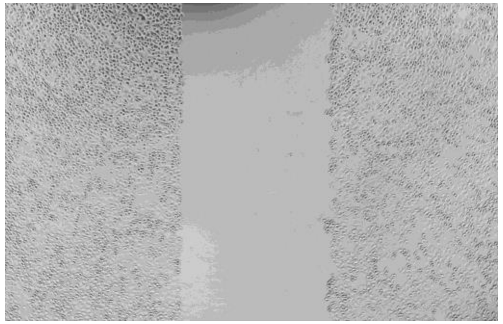

DDP(1)

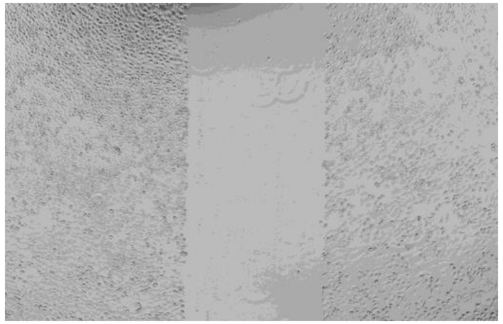

50μM(1)

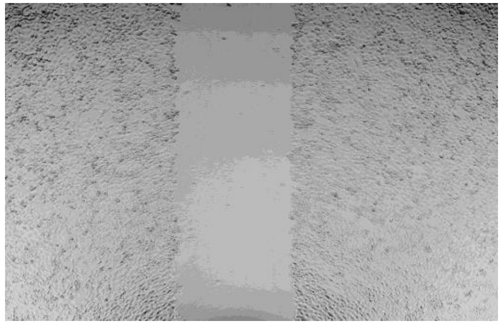

Control(2)

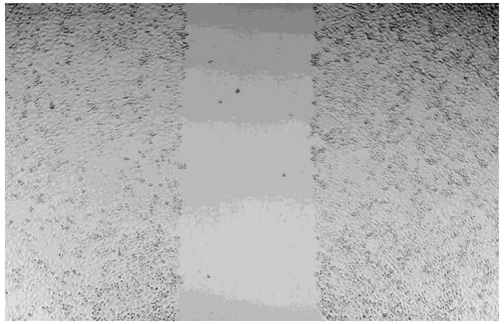

DMSO(2)

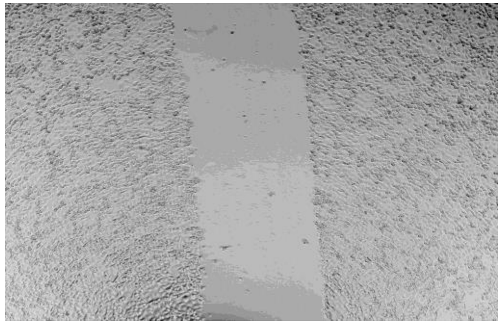

DDP(2)

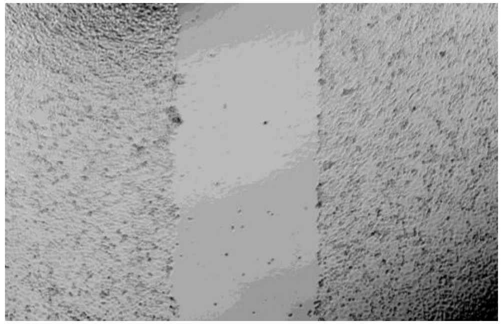

50μM(2)

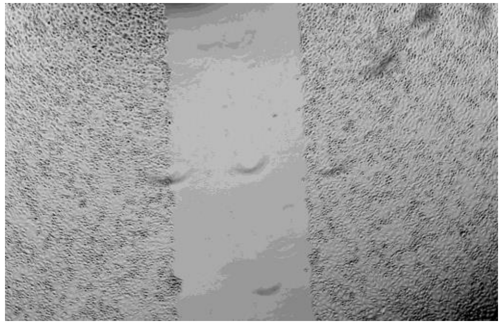

Control(3)

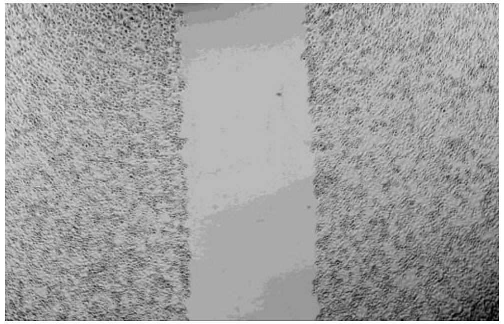

DMSO(3)

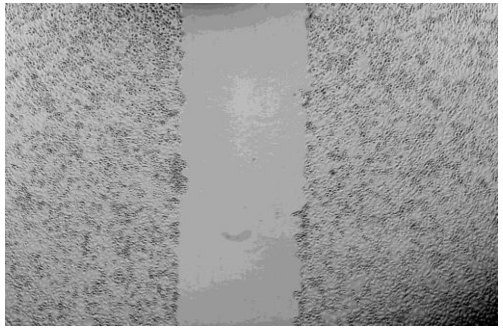

DDP(3)

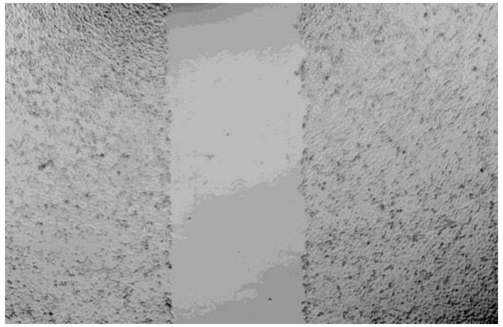

50μM(3)

4.R script

```
if (!requireNamespace("BiocManager", quietly = TRUE))
	install.packages("BiocManager")
BiocManager::install("limma")
install.packages("pheatmap")
logFCfilter=1
adjPfilter=0.05
conNum=58
treatNum=58
library(limma)
setwd("D:\\06.diffAdjPval")
rt=read.table("sampleExp.txt",sep="\t",header=T,check.names=F)
rt=as.matrix(rt)
rownames(rt)=rt[,1]
exp=rt[,2:ncol(rt)]
dimnames=list(rownames(exp),colnames(exp))
rt=matrix(as.numeric(as.matrix(exp)),nrow=nrow(exp),dimnames=dimnames)
rt=avereps(rt)
rt=normalizeBetweenArrays(rt)
Type=c(rep("con",conNum),rep("treat",treatNum))
design <- model.matrix(~0+factor(Type))
colnames(design) <- c("con","treat")
fit <- lmFit(rt,design)
cont.matrix<-makeContrasts(treat-con,levels=design)
fit2 <- contrasts.fit(fit, cont.matrix)
fit2 <- eBayes(fit2)
allDiff=topTable(fit2,adjust='fdr',number=200000)
write.table(allDiff,file="all.xls",sep="\t",quote=F)
diffSig <- allDiff[with(allDiff, (abs(logFC)>logFCfilter & adj.P.Val < adjPfilter )), ]
diffSigOut=rbind(id=colnames(diffSig),diffSig)
write.table(diffSigOut,file="diff.xls",sep="\t",quote=F,col.names=F)
diffUp <- allDiff[with(allDiff, (logFC>logFCfilter & adj.P.Val < adjPfilter )), ]
diffUpOut=rbind(id=colnames(diffUp),diffUp)
write.table(diffUpOut,file="up.xls",sep="\t",quote=F,col.names=F)
diffDown <- allDiff[with(allDiff, (logFC < -logFCfilter & adj.P.Val < adjPfilter )), ]
diffDownOut=rbind(id=colnames(diffDown),diffDown)
write.table(diffDownOut,file="down.xls",sep="\t",quote=F,col.names=F)
library(pheatmap)
geneNum=20
diffSig=diffSig[order(as.numeric(as.vector(diffSig$logFC))),]
diffGeneName=as.vector(rownames(diffSig))
diffLength=length(diffGeneName)
hmGene=c()
if(diffLength>40){
	hmGene=diffGeneName[c(1:geneNum,(diffLength-geneNum+1):diffLength)]
```

```
}else{
    hmGene=diffGeneName
}
hmExp=rt[hmGene,]
Type=c(rep("C",conNum),rep("T",treatNum))
names(Type)=colnames(rt)
Type=as.data.frame(Type)
pdf(file="heatmap.pdf",height=5.5,width=8)
pheatmap(hmExp,
        annotation=Type,
        color = colorRampPalette(c("green", "black", "red"))(50),
        cluster_cols =F,
        show_colnames = F,
        #scale="row",
        fontsize = 10,
        fontsize_row=8,
        fontsize_col=10)
dev.off()

pdf(file="vol.pdf",width=5,height=5)
yMax=max(-log10(allDiff$adj.P.Val))
yMax=ifelse(yMax>100,100,yMax)
xMax=max(abs(allDiff$logFC))
xMax=ifelse(xMax>10,10,xMax)
plot(allDiff$logFC, -log10(allDiff$adj.P.Val), ylab="-log10(adj.P.Val)",xlab="logFC",
    main="Volcano",     ylim=c(0,yMax),xlim=c(-xMax,xMax),yaxs="i",pch=20,
cex=1)
diffSub=subset(allDiff, adj.P.Val<adjPfilter & logFC>logFCfilter)
points(diffSub$logFC, -log10(diffSub$adj.P.Val), pch=20, col="red",cex=1.2)
diffSub=subset(allDiff, adj.P.Val<adjPfilter & logFC<(-logFCfilter))
points(diffSub$logFC, -log10(diffSub$adj.P.Val), pch=20, col="green",cex=1.2)
abline(v=0,lty=2,lwd=3)
dev.off()
```

5.STRING PPI network

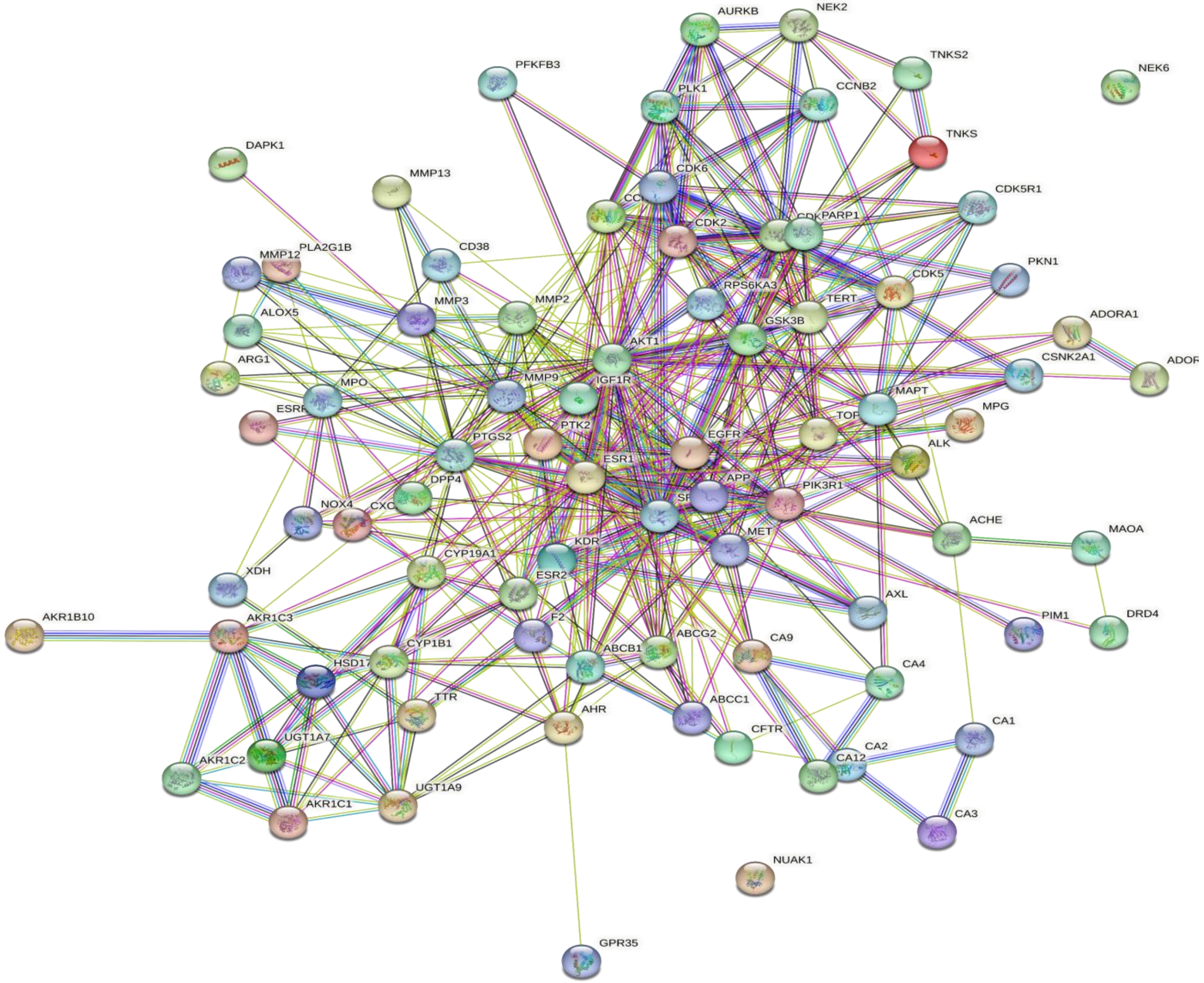

6.Boxplot of mRNA expression of core genes

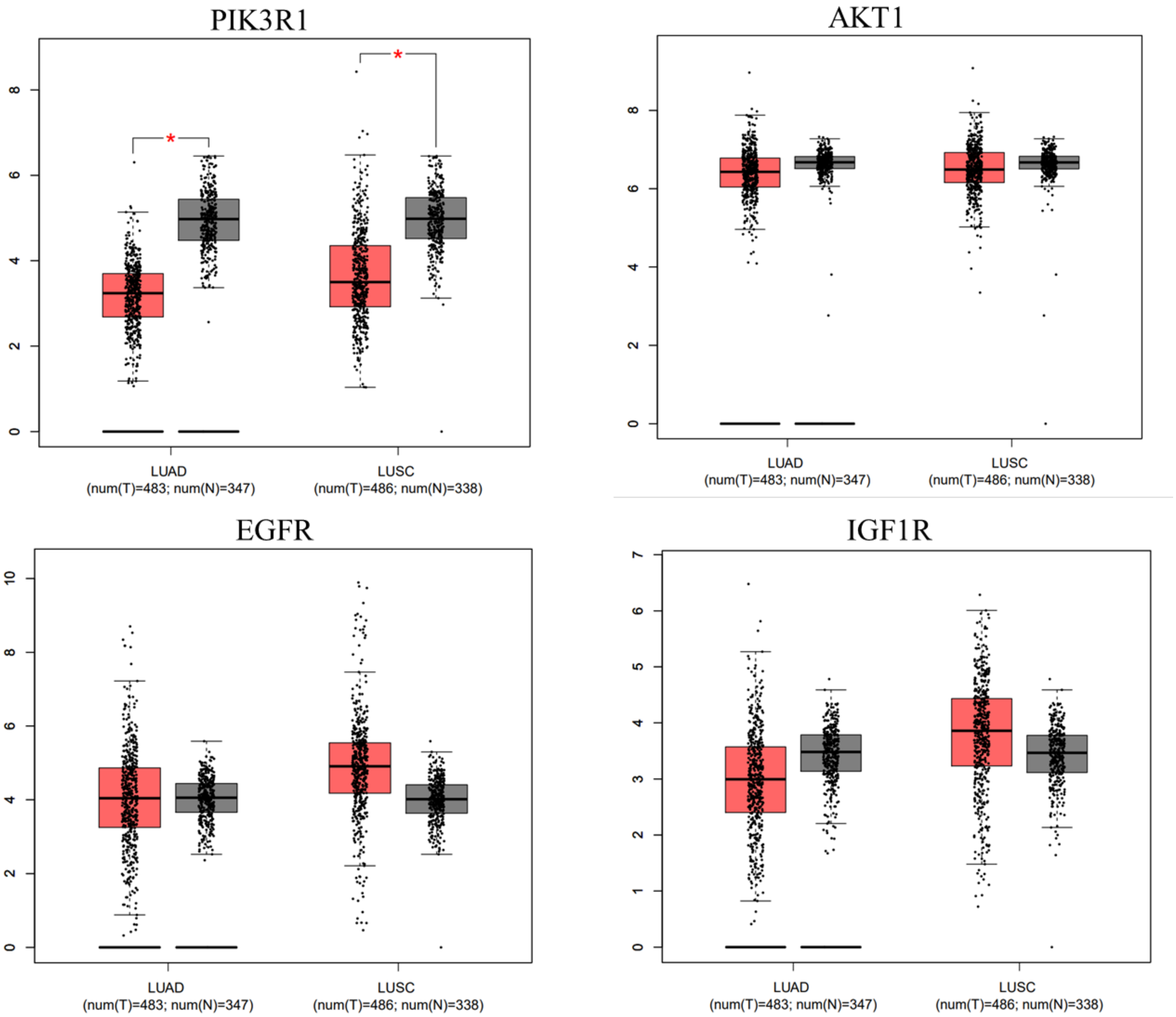

7.The representative protein expression of the core proteins

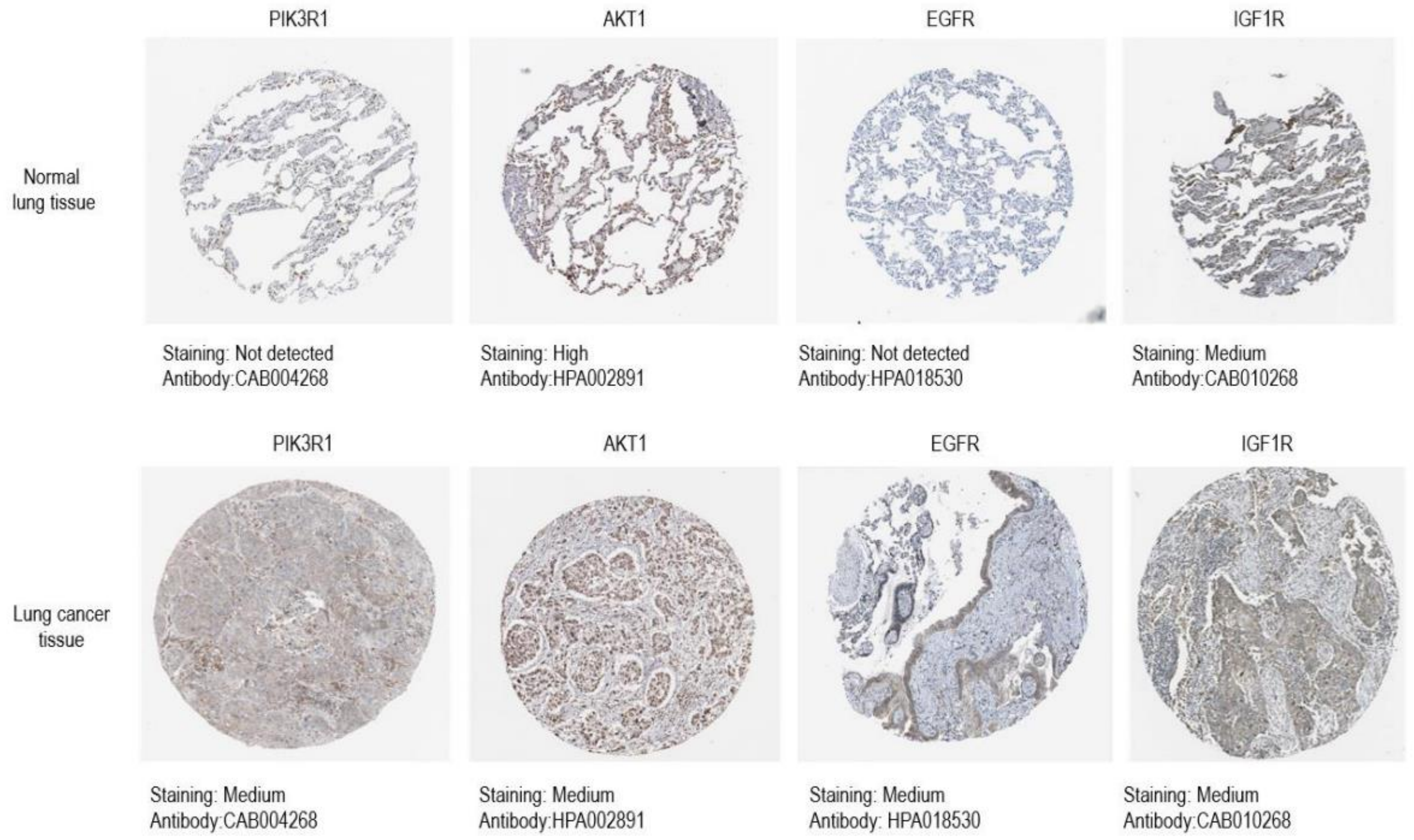

8.The prognostic value of the expression of the 4 hub genes

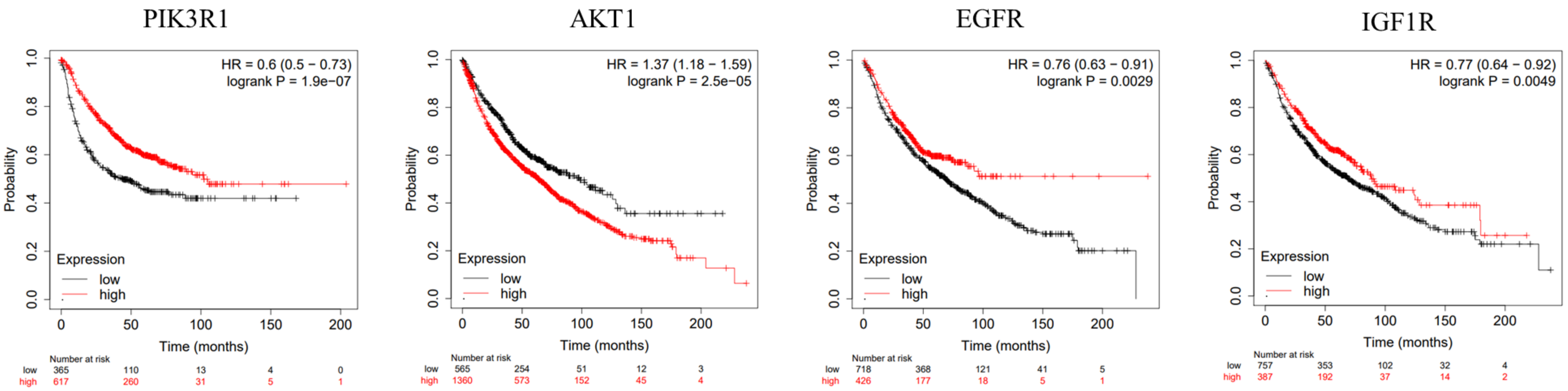

Supplement: Supplementary file 1 [file DataSheet1.pdf]
